# Audiovisual Augmentation of Electronic Consent to Improve Consent Rates and Comprehension: A Randomized Clinical Trial

**DOI:** 10.1001/jamanetworkopen.2026.9347

**Published:** 2026-04-30

**Authors:** Pishoy Gouda, LáShauntá Glover, Aarti Kenjale, Karen Chiswell, Tyler Erickson, Benjamin Goldstein, Michelle Kelsey, Jamie Roberts, Eric D. Peterson, Manesh Patel, George Truskey, Svati Shah, Pamela S. Douglas, Adam J. Nelson, Neha J. Pagidipati

**Affiliations:** 1 Duke Clinical Research Institute, Durham, North Carolina; 2 University of Alberta, Edmonton, Alberta, Canada; 3 Department of Population Health Science, Duke University School of Medicine, Durham, North Carolina; 4 Department of Medicine, Duke University, Durham, North Carolina; 5 Duke University Medical Center, Durham, North Carolina; 6 University of Texas Southwestern Medical School, Dallas; 7 University of Adelaide, Adelaide, Australia

## Abstract

**Importance:**

The use of audiovisual aids in the consent process may improve comprehension and willingness to participate in research. However, data supporting this hypothesis are lacking.

**Objective:**

To determine the impact of audiovisual augmentation of the consent process on willingness to participate in research and on consent comprehension.

**Design, Setting, and Participants:**

The Personalizing Cardiovascular Health: A Population Approach to Promoting Cardiovascular Disease Resistance and Resilience Among Individuals With Obesity (RESILIENCE) nonrandomized cohort study embedded this multiarm randomized clinical trial to test different participant consent modalities. RESILIENCE recruited participants between September 2019 and March 2022, with the analysis for this randomized substudy conducted from July to December 2025. No follow-up was undertaken for this study. This component of the study was conducted exclusively virtually and completed online within a single large US academic health system.

**Interventions:**

Potential participants were randomized for this trial in a 1:1:1:1 allocation to receive consent information via (1) text-only (2) text and physician-featured video, (3) text and patient-featured video, or (4) text and animated video.

**Main Outcomes and Measures:**

The primary outcome was provision of consent to participate in the cohort clinical study. The secondary outcome was consent comprehension, based on a score of at least 5 of 7 on a follow-up comprehension survey. Robust log-linear Poisson regression was used to determine the relative risks (RRs) of outcomes in comparison with the text-only arm. Analyses were conducted with the evaluable population.

**Results:**

Of 1535 participants (968 [63.1%] female; 658 [42.9%] ≥60 years of age; 380 randomized to text-only, 386 to text and physician-featured video, 383 to text and patient-featured video, and 386 to text and animated video) who began the consent process, 888 (57.9%) overall gave consent to participate in the study. Patient consent rates were similar across the 4 consent delivery strategies (text only [comparator], 221 of 380 [58.2%]; text and physician video, 241 of 386 [62.4%]; RR, 1.07 [96% CI, 0.95-1.21]; text and patient video, 203 of 383 [53.0%]; RR, 0.91 [96% CI, 0.8-1.04]; and text and animated video, 223 of 386 [57.8%]; RR, 0.99 [96% CI, 0.88-1.13]). Among 884 individuals who consented and completed the consent comprehension survey, the rate of comprehension of the consent process was high 86.4% (n = 764) and did not vary among the intervention delivery strategies (text only, 193 of 221 [87.3%]; text and physician video, 207 of 241 [85.9%]; text and patient video, 177 of 203 [87.2%]; and text and animated video, 187 of 223 [83.9%]). Compared with the text-only arm, the RRs of consent comprehension were not different for the text and physician-featured video arm (RR, 1.00; [95% CI, 0.93-1.07]), the text and patient-featured video arm (RR, 1.00 [95% CI, 0.93-1.07]) and the text and animated video arm (RR, 0.96 [95% CI, 0.89-1.04]).

**Conclusions and Relevance:**

In this randomized clinical trial of virtual consent delivery, the rates of patient consent and consent comprehension were similar regardless of whether they were delivered by text only or text augmented by physician, patient, or animated videos. Further evaluation of these consent delivery processes across different study types and patient populations is suggested to optimize the recruitment of diverse, informed populations.

**Trial Registration:**

ClinicalTrials.gov Identifier: NCT04551872

## Key Points

**Question:**

Does adding audiovisual content to text-only electronic consent improve participant consent rates or comprehension compared with standard text-only consent?

**Findings:**

In this randomized clinical trial including 1535 participants, consent rates were similar across text-only, text plus physician video, text plus patient video, and text plus animated video groups, with no statistically significant differences. Among 884 participants who consented, overall comprehension of the consent process was high (86%) and did not differ significantly by consent strategy.

**Meaning:**

Audiovisual augmentation of electronic consent did not improve consent uptake or understanding over text-only consent.

## Teaser

This randomized clinical trial assesses whether including audiovisual content featuring a physician, patient, or animation with standard text-only electronic consent improves participant consent rates or comprehension compared with text only.

## Introduction

Informed consent is a critical part of the research process, ensuring that participants are aware of the potential risks and benefits of a study and are entering into a study fully aware of the information needed to make an informed decision to participate. This process is centered around the principles of autonomy, beneficence, and justice.^1^ Informed consent traditionally includes a description of the proposed research, the basis for participant selection, potential risks and benefits, confidentiality, and participant rights. However, many studies have demonstrated that in both the clinical and research context, consent is only infrequently informed, with many studies showing that after the traditional consent process, patients poorly comprehend the information shared with them.^2,3,4,5,6,7,8,9,10^ As a result, there has been considerable focus on improving the informed consent process with audiovisual aids.^11,12,13^

Previous studies exploring the results of an audiovisual augmented consent experience are limited by nonrandomized design, small sample sizes, or a hypothetical research study setting.^14^ The use of electronic devices to deliver and receive informed consent (e-consent) has facilitated the provision of an audiovisual augmented consent process. This approach has several advantages, including increased accessibility, the possibility of increased engagement and comprehension, and increased participant satisfaction with the process.^15^

The Personalizing Cardiovascular Health: A Population Approach to Promoting Cardiovascular Disease Resistance and Resilience Among Individuals With Obesity (RESILIENCE) cohort study was designed with an embedded multiarm randomized clinical trial. The goal of this trial was to explore the effects of various audiovisual augmented e-consent strategies.

## Methods

### RESILIENCE Study

In brief, RESILIENCE was a prospective cohort study conducted in the Duke University Health System between September 2019 and March 2022. All participants provided informed consent, and the study received approval from the Duke institutional review board. The present embedded randomized clinical trial was reported per the Consolidated Standards of Reporting Trials (CONSORT) and Studies Within a Trial (SWATs) reporting guidelines.^16,17^The trial protocol is available in Supplement 1.

The RESILIENCE study recruited participants across the spectrum of body mass index (BMI, calculated as weight in kilograms divided by height in meters squared) and cardiovascular risk who underwent clinical, behavioral, and biological assessment to explore the interplay between obesity, atherosclerotic cardiovascular disease (ASCVD) risk, and weight loss after a virtual health behavioral intervention. Recruited participants included (1) individuals with a BMI of at least 30 and a 10-year ASCVD risk of at least 20%, (2) individuals with a BMI of at least 30 and a 10-year ASCVD risk of 7.5% or lower, (3) individuals with a BMI of 18 to 25 and a 10-year ASCVD risk of at least 20%, and (4) individuals with a BMI of 18 to 25 and a 10-year ASCVD risk lower than 7.5%.

The RESILIENCE cohort study included 2 embedded randomized clinical trials that explored the impact of various methods of potential participant recruitment and various methods of consent. In 1 of the substudies, potential participants were identified through electronic medical records (EMRs) based on the RESILIENCE study eligibility criteria. Participants were required to have internet access, an email address, access to the EMR patient portal, access to a smart phone, and the ability to read and understand English. Participants were randomized in a factorial manner to a method of contact (EMR patient portal vs email) and message content (altruistic vs individualistic messaging). The primary outcome was clicking on the embedded link in the EMR message or email and landing on the study website.

### Methods of Consent Randomized Trial Design

In the present randomized clinical trial substudy, once potential participants landed on the study website, they were randomized to receive 1 of 4 consent strategies: (1) text-based consent (11 pages), which is considered standard for this type of study, (2) text-based plus video consent with the information provided by a patient, (3) text-based plus video consent with information provided by a study physician, or (4) text-based plus video consent with information provided in the form of animation (Supplement 1). Each of the videos was approximately 13.5 minutes and followed the same content script (grade 8 literacy). The patient video included an actor, who was a middle-aged African American woman, and the physician video was recorded by a young female physician from a visible racial and ethnic minority group. The content of these messages was crafted by study investigators through iterative feedback from a patient advisory group, which consisted of 6 individuals with lived experiences relevant to the study, with an emphasis on tailoring the information to underrepresented or minoritized populations. All content was created by professional graphic designers and videographers.

Participants who provided consent to proceed with the study then answered a 7-question consent comprehension survey exploring their understanding of the consent process at the end of the encounter (eTable in Supplement 2). The primary end point was providing consent to participate in the RESILIENCE study. The secondary end point was participant comprehension of the consent process, which was categorized as a score of 5 or higher of a total possible score of 7 on the assessment following the consent process. The 7-question consent comprehension survey was developed to assess components that are integral to the consent process, including autonomy, voluntariness, access to information, opportunity to ask questions, understanding of study procedures, understanding of participant responsibilities, and understanding the right to withdraw and confidentiality. Questions were developed and refined based on iterative feedback from the study team (L.G., A.K., J.R., and N.P.) and patient partners after review of consent content. Subgroups of interest included ages younger than 60 years and 60 years or older, sex, race, and ASCVD-BMI risk phenotype. Race was collected in the study to more fully characterize the cohort in accordance with the American Medical Association guidelines. Participants selected from the following race and ethnicity categories: American Indian or Alaskan native, Asian, Black or African American, White, or a combination of 2 or more races. By the nature of the study, participants were not blinded to the treatment group. Both randomization and outcomes occurred before the involvement of the research team, negating the need for blinding.

### Statistical Analysis

Descriptive statistics were used to summarize continuous variables, reported as medians with IQRs. Categorial variables were described as absolute numbers and percentages. The primary end point (providing consent) was dichotomized as yes or no and reported as an absolute number and percentage in each randomization arm. The secondary end point (consent comprehension) was assessed among participants who consented and was dichotomized as yes or no, with a score of at least 5 on the survey categorized as yes. Robust log-linear Poisson regression models were used to estimate model-based relative risks (RRs) of consenting and consent comprehension. Models were fit using generalized estimating equations with robust sandwich variance estimators and included treatment group, subgroup, and treatment-by-subgroup interaction terms. As each participant contributed a single binary outcome, models used an independence working correlation structure. Generalized estimating equations were used as a convenient framework to obtain robust (sandwich) standard errors for log-link Poisson models estimating risk ratios, rather than to model within-participant repeated measures. For the primary outcome, the global null hypothesis of no differences between randomization groups was tested, and pairwise differences between randomization groups were quantified using RRs and the associated 96% CIs, with the text-only group as the reference category. For the secondary outcome, the null hypothesis of no differences between randomization groups was tested, and pairwise differences between randomization groups were quantified using RRs and the associated 95% CIs, with the text-only group as the reference category. Sample size calculations were not determined for this study but were based on the primary study requirements.

A single prespecified interim analysis of the primary end point was conducted to evaluate whether any consenting strategy appeared substantially inferior and should be discontinued. To preserve the overall type I error rate of .05, a conservative 2-sided α level of .01 was allocated to the interim analysis, with the remaining α of .04 reserved for the final analysis. This approach represents a simple α-spending strategy rather than a formal group-sequential design. At the interim analysis, no consenting strategy demonstrated a meaningfully lower consent rate compared with the others. As a result, no study arms were discontinued and the trial continued as originally designed. A global 3 degrees of freedom hypothesis test of any difference between the 4 consenting strategies was tested at an α of .04. Multiplicity was not controlled between the primary and secondary end point analyses, with the secondary end point reported with an α of .05 and effect estimates with a nominal 95% CI. Subgroup analyses within the primary and secondary end points should be considered exploratory and are reported with 95% CIs. Missing data were excluded from all denominators, with no imputation undertaken for missing data. All analyses were conducted with the evaluable population using SAS, version 9.4, (SAS Institute Inc).

## Results

### Baseline Characteristics

Between September 2019 and March 2022, a total of 1535 participants began the online consent process; 380 (24.8%) were randomized to the text-only arm, 386 (25.1%) to the text and physician video arm, 383 (25.0%) to the text and patient video arm, and 386 (25.1%) to the text and animated video arm (Figure). Overall, 968 (63.1%) were female, 567 (36.9%) were male, and 658 (42.9%) were at least 60 years of age. In addition, 39 (2.5%) were Asian, 309 (20.1%) were Black or African American, 1130 (73.6%) were White, and 28 (1.8%) were a combination of 2 or more races (Table 1). A history of hypertension requiring treatment was present in 769 (50.2%) of participants, of diabetes in 381 (24.8%), and of smoking in 80 (5.2%). Based on the risk phenotype, 541 (35.2%) had an elevated BMI with a high ASCVD risk, 454 (29.6%) had an elevated BMI with a low ASCVD risk, 180 (11.7%) had a normal BMI with a high ASCVD risk, and 360 (23.5%) had a normal BMI with a low ASCVD risk.

**Figure.**
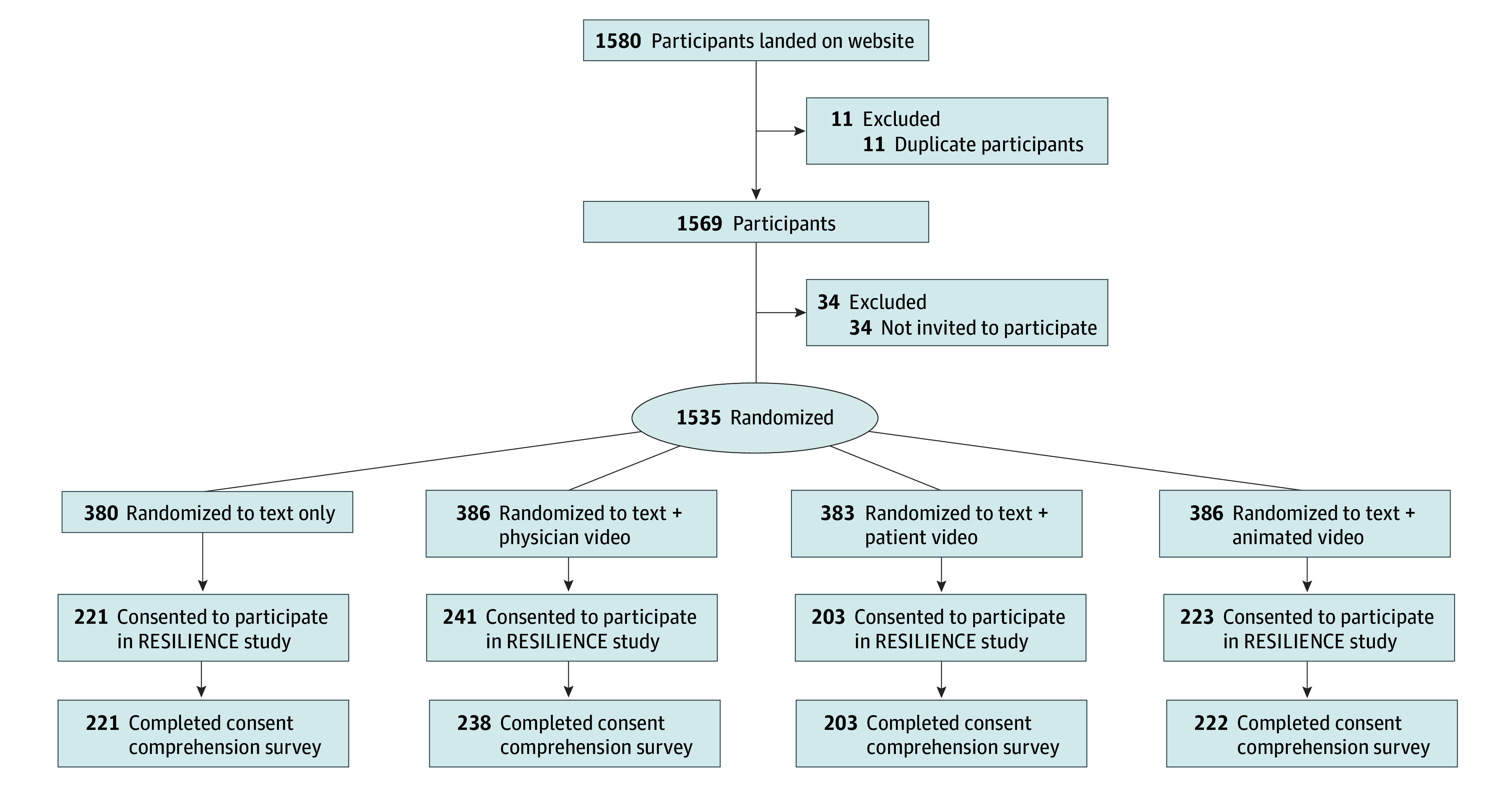
CONSORT Diagram CONSORT indicates Consolidated Standards of Reporting Trials; RESILIENCE, The Personalizing Cardiovascular Health: A Population Approach to Promoting Cardiovascular Disease Resistance and Resilience Among Individuals With Obesity study.

**Table 1.** Baseline Demographics by Randomized Consent Group

Baseline characteristic	Participants, No. (%)				
	All participants (n = 1535)	Text and physician video (n = 386)	Text and patient video (n = 383)	Text and animated video (n = 386)	Text only (n = 380)
Age group, y					
≥60	658 (42.9)	167 (43.3)	164 (42.8)	166 (43.0)	161 (42.4)
<60	877 (57.1)	219 (56.7)	219 (57.2)	220 (57.0)	219 (57.6)
Sex					
Female	968 (63.1)	239 (61.9)	231 (60.3)	255 (66.1)	243 (63.9)
Male	567 (36.9)	147 (38.1)	152 (39.7)	131 (33.9)	137 (36.1)
Race					
Black or African American	309 (20.1)	79 (20.5)	82 (21.4)	84 (21.8)	64 (16.8)
White	1130 (73.6)	278 (72.0)	277 (72.3)	277 (71.8)	298 (78.4)
Other^a^	70 (4.6)	19 (4.9)	18 (4.7)	20 (5.2)	13 (3.4)
Not reported or declined to report	26 (1.7)	10 (2.6)	6 (1.6)	5 (1.3)	5 (1.3)
BMI, median (IQR)^b^	31.8 (23.8-36.4)	32.2 (23.7-36.8)	31.8 (23.6-36.2)	31.7 (23.9-36.2)	31.9 (24.2-36.4)
Study phenotype					
Obesity high risk	541 (35.2)	138 (35.8)	140 (36.6)	138 (35.8)	125 (32.9)
Obesity low risk	454 (29.6)	116 (30.1)	107 (27.9)	109 (28.2)	122 (32.1)
Nonobesity high risk	180 (11.7)	44 (11.4)	38 (9.9)	49 (12.7)	49 (12.9)
Nonobesity low risk	360 (23.5)	88 (22.8)	98 (25.6)	90 (23.3)	84 (22.1)
Comorbidity or risk factor					
Diabetes	381 (24.8)	100 (25.9)	92 (24.0)	92 (23.8)	97 (25.5)
Smoker	80 (5.2)	24 (6.2)	18 (4.7)	22 (5.7)	16 (4.2)
Treated for hypertension	769 (50.2)	188 (48.8)	177 (46.3)	204 (52.8)	200 (52.8)

Abbreviation: BMI, body mass index.

^a^ Other races included American Indian or Alaskan native (n = 3), Asian (n = 39), and a combination of 2 or more races (n = 28).

^b^ BMI calculated as weight in kilograms divided by height in meters squared.

### Primary Outcome

The primary outcome (provision of consent for study) was assessed among all 1535 participants randomized. The primary end point occurred in 221 of 380 participants (58.2%) in the text-only arm, 241 of 386 participants (62.4%) in the text plus physician video arm, 223 of 386 participants (57.8%) in the text and animated video arm, and 203 of 383 participants (53.0%) in the text and patient video arm, with no significant differences between groups (*P* = .07; Figure). In comparison with the text-only arm, the RRs of consent were not different in the text and physician video arm (RR, 1.07 [96% CI, 0.95-1.21]; *P* = .07), the text and patient video arm (RR, 0.91 [96% CI, 0.80-1.04]) or the text and animated video arm (RR, 0.99 [96% CI, 0.88-1.13]). No significant heterogeneity in effect was observed by age group (interaction *P* = .49), race (interaction *P* = .30), sex (interaction *P* = .67) or risk phenotype (interaction *P* = .20; Table 2). A higher percentage of Black or African American individuals provided consent in the text plus patient video group compared with the text only group, but the difference was not statistically significant (72.2% vs 53.1%; RR, 1.36 [95% CI, 1.04-1.78]; *P* = .07).

**Table 2.** Primary Outcome (Provision of Consent) Stratified by Consent Group

Characteristic	Text only, reference group (n = 380), No./total No. (%)	Text and physician video (n = 386)		Text and patient video (n = 383)		Text and animated video (n = 386)		Global interaction *P* value^a^
		No./total No. (%)	RR (95 CI%)	No./total No. (%)	RR (95 CI%)	No./total No. (%)	RR (95 CI%)	
Age group, y								
≥60	90/161 (55.9)	108/167 (64.7)	1.02 (0.87-1.18)	81/164 (49.4)	0.93 (0.79-1.09)	95/166 (57.2)	0.97 (0.83-1.14)	.49
<60	131/219 (59.8)	133/219 (60.7)	1.16 (0.97-1.38)	122/219 (55.7)	0.88 (0.72-1.09)	128/220 (58.2)	1.02 (0.85-1.24)	
Sex								.67
Female	143/243 (58.8)	147/239 (61.5)	1.05 (0.90-1.21)	121/231 (52.4)	0.89 (0.76-1.05)	141/255 (55.3)	0.94 (0.81-1.09)	
Male	78/137 (56.9)	94/147 (63.9)	1.12 (0.93-1.36)	82/152 (53.9)	0.95 (0.77-1.17)	82/131 (62.6)	1.1 (0.90-1.34)	
Race								
Black or African American	34/64 (53.1)	57/79 (72.2)	1.36 (1.04-1.78)	50/82 (61.0)	1.15 (0.86-1.53)	57/84 (67.9)	1.28 (0.97-1.68)	.30
White	175/298 (58.7)	165/278 (59.4)	1.01 (0.88-1.16)	143/277 (51.6)	0.88 (0.76-1.02)	153/277 (55.2)	0.94 (0.82-1.08)	
Other^b^	9/13 (69.2)	13/19 (68.4)	0.98 (0.65-1.50)	8/18 (44.4)	0.63 (0.35-1.11)	8/20 (40.0)	0.78 (0.47-1.28)	
Study phenotype								
Obesity high risk	72/125 (57.6)	91/138 (65.9)	1.14 (0.94-1.39)	74/140 (52.9)	0.92 (0.74-1.14)	85/138 (61.6)	1.07 (0.88-1.31)	.20
Obesity low risk	78/122 (63.9)	86/116 (74.1)	1.13 (0.75-1.71)	64/107 (59.8)	0.94 (0.76-1.15)	75/109 (68.8)	1.08 (0.90-1.29)	
Nonobesity high risk	34/49 (69.4)	26/44 (59.1)	0.85 (0.63-1.16)	30/38 (78.9)	1.14 (0.89-1.46)	30/49 (61.2)	0.88 (0.66-1.18)	
Nonobesity low risk	37/84 (44.0)	38/88 (43.2)	0.98 (0.70-1.38)	35/98 (35.7)	0.81 (0.57-1.16)	33/90 (36.7)	0.83 (0.58-1.20)	

Abbreviation: RR, relative risk.

^a^ RRs estimated using robust log-linear Poisson regression models fit with generalized estimating equations, with the text-only treatment arm as the comparator. RRs are model-based estimates derived from treatment by subgroup interaction models and do not necessarily equal crude ratios calculated directly from the cell proportions.

^b^ Other races included American Indian or Alaskan native (n = 3), Asian (n = 39), and a combination of 2 or more races (n = 28).

### Consent Comprehension

Of the 888 participants who provided consent to proceed with the study, 4 participants did not complete the survey and were excluded from the comprehension analysis. Of the remaining 884 participants, consent comprehension was high overall, with 764 (86.4%) achieving a score of at least 5 of 7. The rates of consent comprehension were similar across randomization groups (text only, 193 of 221 [87.3%]; text and physician video, 207 of 241 [85.9%]; text and patient video, 177 of 203 [87.2%]; and text and animated video, 187 of 223 [83.9%]). In comparison with the text-only arm, the RRs of consent comprehension were not different for the text and physician video arm (RR, 1.00 [95% CI, 0.93-1.07]), the text and patient video arm (RR, 1.00 [95% CI, 0.93-1.07]) and the text and animated video arm (RR, 0.96 [95% CI, 0.89-1.04]). No significant heterogeneity in effect was observed by age group (interaction *P* = .66), race and ethnicity (interaction *P* = .37), sex (interaction *P* = .33) or risk phenotype (interaction *P* = .72) (Table 3).

**Table 3.** Secondary Outcome (Consent Comprehension) Stratified by Consent Group

Characteristic	Text only, reference group (n = 221), No./total No. (%)	Text and physician video (n = 238)		Text and patient video (n = 203)		Text and animated video (n = 222)		Global interaction *P* value^a^
		No./total No. (%)	RR (95% CI)	No./total No. (%)	RR (95% CI)	No./total No. (%)	RR (95% CI)	
Age group, y								
≥60	76/90 (84.4)	93/105 (88.6)	0.96 (0.88-1.05)	71/81 (87.7)	0.97 (0.89-1.07)	79/95 (83.2)	0.95 (0.87-1.05)	.66
<60	117/131 (89.3)	114/133 (85.7)	1.05 (0.94-1.17)	106/122 (86.9)	1.04 (0.92-1.17)	108/127 (85.0)	0.98 (0.87-1.12)	
Sex								
Female	120/143 (83.9)	125/145 (86.2)	1.03 (0.93-1.13)	103/121 (85.1)	1.01 (0.91-1.12)	119/140 (85.0)	1.01 (0.92-1.12)	.33
Male	73/78 (93.6)	82/93 (88.2)	0.94 (0.86-1.04)	74/82 (90.2)	0.96 (0.88-1.06)	68/82 (82.9)	0.89 (0.79-0.99)	
Race								
Black or African American	26/34 (76.5)	48/56 (85.7)	1.12 (0.90-1.39)	40/50 (80.0)	1.05 (0.83-1.32)	45/57 (78.9)	1.03 (0.82-1.30)	.37
White	156/175 (89.1)	146/163 (89.6)	1 (0.93-1.08)	128/143 (89.5)	1 (0.93-1.08)	134/152 (88.2)	0.99 (0.91-1.07)	
Other^b^	8/9 (88.9)	9/13 (69.2)	0.75 (0.53-1.06)	7/8 (87.5)	0.98 (0.75-1.28)	5/8 (62.5)	0.67 (0.42-1.07)	
Study phenotype								
Obesity high risk	61/72 (84.7)	80/91 (87.9)	1.04 (0.92-1.17)	63/74 (85.1)	1 (0.88-1.15)	68/84 (81.0)	0.96 (0.83-1.10)	.72
Obesity low risk	65/78 (83.3)	70/83 (84.3)	1.03 (0.84-1.26)	56/64 (87.5)	1.05 (0.92-1.20)	63/75 (84.0)	1.01 (0.88-1.16)	
Nonobesity high risk	36/37 (97.3)	23/26 (88.5)	0.97 (0.82-1.15)	26/30 (86.7)	0.95 (0.80-1.13)	28/30 (93.3)	1.02 (0.89-1.18)	
Nonobesity low risk	31/34 (91.2)	34/38 (89.5)	0.92 (0.81-1.04)	32/35 (91.4)	0.94 (0.84-1.05)	28/33 (84.8)	0.87 (0.75-1.02)	

Abbreviation: RR, relative risk.

^a^ RRs estimated using robust log-linear Poisson regression models fit with generalized estimating equations, with the text-only treatment arm as the comparator. RRs are model-based estimates derived from treatment by subgroup interaction models and do not necessarily equal crude ratios calculated directly from the cell proportions.

^b^ Other races included American Indian or Alaskan native (n = 3), Asian (n = 39), and a combination of 2 or more races (n = 28).

## Discussion

In this randomized clinical trial comparing the effectiveness of 4 e-consent strategies embedded in a cohort study assessing individuals with ASCVD risk and obesity, we demonstrated high overall rates of consent (57.9%) and comprehension of the consent process (86.4%) using an e-consent strategy. However, augmenting the consent process with the use of audiovisual aids of various formats was not associated with either higher rates of consent or consent comprehension when compared with a traditional text-only format.

There are few large, randomized studies exploring the use of audiovisual augmented informed consent for research studies. In a trial exploring the utility of telehealth in the management of chronic obstructive pulmonary disease, 4124 potential participants were randomized to receive a traditional recruitment letter in the mail or the addition of a link to additional multimedia resources describing the study, generic information about research participation, and bespoke information about the benefits of participation.^18^ Similarly, in a gout pharmaceutical trial, 509 potential participants were randomized to receive traditional recruitment material or the addition of a DVD that described the background of the study and operational aspects of the trial.^19^ Two other trials examined the utility of asking oncology patients (n = 145 and n = 173) to watch a video about clinical trials in general or a specific clinical trial prior to their clinic appointment.^20,21^ However, these studies were heterogeneous in their nature and use of audiovisual content, making it challenging to compare these studies head to head due to variations in the nature of the studies, invasiveness of intervention, relative risks and benefits, required commitment from participants, and setting. Importantly, as in our study, the use of an audiovisual component in the consent process did not increase the rate of consent across any of these aforementioned studies.^18,19,20,21^ Overall, randomized trials of audiovisual consent processes have not identified any harm with their use and found that they may be associated with increased participant knowledge, reduced anxiety, and higher participant satisfaction with the process.^14,20,22,23,24^ As far as we are aware, no other trials have assessed an audiovisual consent process in a purely virtual setting.

It is important to recognize that there are cultural and contextual factors that may influence decision-making during the consent process, with trust being an important factor in many racial and ethnic minority groups.^25^ While hypothesis generating, our data support this concept, with Black or African American individuals more frequently providing consent in the physician video arm. Further exploration of optimal consent methods across race and ethnicity is required.

An important consideration when evaluating audiovisual aids is to understand that they are not all created equally, with a spectrum of interactivity that lends itself well to modern audiovisual technology. This is illustrated in a 2 by 2 factorial design randomized clinical trial of recruitment methods to enroll participants in a Biobank study.^26^ Participants were randomized to either an in-person or multimedia approach and to either a standard or enhanced interactivity approach. The increased interactivity took the form of a series of 13 interactive questions, which were delivered in person or via the multimedia tool. The results demonstrated that both the multimedia and the increased interactivity components were independently associated with increased participant understanding.^26^

Our findings highlight that incorporating audiovisual components into the consent process requires substantial resources, including the development and customization of content for a specific study. Although video-based consent has the potential to be more engaging and digestible than text alone, the 13-minute duration of the videos used in this study may itself have limited their usability and impact. Shorter, more focused, and modular video segments may better leverage the advantages of audiovisual media and enhance participant engagement in future e-consent platforms. Importantly, any such approach must balance brevity with the need to provide sufficient information to ensure truly informed consent. In the present study, the investment in audiovisual content did not translate into higher consent rates. However, these findings may not be generalizable to all study contexts, particularly those involving more complex interventions, such as gene therapies or surgical procedures, in which audiovisual aids may offer greater benefit.

### Limitations

Several limitations should be considered when interpreting our study. Our study examined consent methodologies in a single study that was minimally invasive and had high rates of consent provision, limiting both generalizability and ability to further improve rates of consent provision. As this analysis of consent comprehension was restricted to participants who voluntarily provided consent, these findings may be subject to self-selection bias and may not be generalizable to individuals who chose not to engage with or complete the consent process. While we demonstrated that consent comprehension was high among individuals who provided consent to be enrolled in the study, by the nature of the study design, we were unable to explore consent comprehension among those who did not provide consent.

As previously stated, the heterogeneous nature of trials and potential participants limits the generalizability of our findings to other studies and participant demographics. This limitation is evidenced by prior studies demonstrating that the effectiveness of recruitment strategies varies by rurality,^27^ age,^28^ and race and ethnicity.^29^ Lastly, our study was conducted during the COVID-19 pandemic, which may have led to changes in behavior of potential research participants due to an increased awareness of their health status and changes in trust with health professionals. Also noteworthy is that no 2 audiovisual consent platforms are identical, which can vary in delivery mechanism and the degree of interaction with the participant, limiting our ability to easily compare the present study with others. As such, the inclusion of different content or in a different format may alter the utility of an audiovisual augmented consent process. For instance, the individuals portrayed in the videos were from a visible minority, and our content was tailored to individuals underrepresented in research. We also observed a higher rate of consent provision among Black or African American individuals. Whether these choices made during content creation influence the consent process is not clear and should be evaluated in subsequent studies. This finding highlights the importance of involving a wide variety of trial team members and potential participants early in the design of such material. Additionally, there is no single gold standard tool that has been developed to assess patient understanding of consent, with many variations used by different studies.^14^

## Conclusions

In this randomized clinical trial of potential research participants, audiovisual augmentation of the text-only e-consent process was not associated with increased participant consent rates or comprehension of the consent content in comparison with the traditional text-based e-consent format. Whether future iterations of this technology have a role in improving the consent experience for research participants will require further research.

## Notes

Supplement 1.
Trial Protocol and Statistical Analysis

Supplement 2. **eTable.** Consent Comprehension Survey

Supplement 3.
Data Sharing Statement
