# Supplementary material for: Audiovisual Augmentation of Electronic Consent to Improve Consent Rates and Comprehension: A Randomized Clinical Trial
Source: JAMA Netw Open. 2026 Apr 30;9(4):e269347. doi: 10.1001/jamanetworkopen.2026.9347 (PMC13133689; doi:10.1001/jamanetworkopen.2026.9347)
Supplement: Supplement 2. — eTable. Consent Comprehension Survey [file jamanetwopen-e269347-s002.pdf]

Supplementary Online Content

Gouda P, Glover L, Kenjale A, et al. Audiovisual augmentation of electronic consent to improve consent rates and comprehension: a randomized clinical trial. *JAMA Netw Open*. 2026;9(4):e269347. doi:10.1001/jamanetworkopen.2026.9347

eTable. Consent Comprehension Survey

This supplementary material has been provided by the authors to give readers additional information about their work.

© 2026 Gouda P et al. *JAMA Network Open*.

eTable. Consent Comprehension Survey

Question	Potential Answer Values (Correct Response in Bold)
Joining the Study is:	a. Required by my personal physician<br>b. Completely voluntary<br>c. Will not affect the care that I get as a patient in the "Institution"<br>**d. Both (b) and (c)**
If I have questions or concerns about joining this study, before I decide I should:	a. Talk to my family and friends<br>b. Talk to the study team or study doctor<br>c. Talk to my personal doctor<br>**d. Any or all of the above**
In this study, one of the things I will do is:	**a. Fill out surveys about my health**<br>b. Take medications that are given to me by the study<br>c. Come to exercise classes at a "institution" gym
To stay in the study, I need to send my samples back to the study team as soon as possible	**a. True**<br>b. False
The results of my genetic tests results will be returned to me whether I want them or not	a. True<br>**b. False**
Clinical laboratory test results:	a. Will be available for me to see in my MyChart (electronic medical record patient portal)<br>b. Will be shared with me and my personal doctor if they show a major risk of a life-threatening or serious health condition<br>c. Will be returned to me on the same day of the test, regardless of the result<br>**d. Both (a) and (b)**
At any point during this study, I can:	a. Stop being in the study<br>b. Speak with the study doctor about questions or concerns that I have<br>c. Expect my personal information to be kept confidential<br>**d. All of the above**

© 2026 Gouda P et al. *JAMA Network Open*.
