# Supplementary material for: Audiovisual Augmentation of Electronic Consent to Improve Consent Rates and Comprehension: A Randomized Clinical Trial
Source: JAMA Netw Open. 2026 Apr 30;9(4):e269347. doi: 10.1001/jamanetworkopen.2026.9347 (PMC13133689; doi:10.1001/jamanetworkopen.2026.9347)
Supplement: Supplement 3. — Data Sharing Statement [file jamanetwopen-e269347-s003.pdf]

Data Sharing Statement

Gouda. Audiovisual Augmentation of Electronic Consent to Improve Consent Rates and Comprehension. *JAMA Netw Open*. Published April 29, 2026.
doi:10.1001/jamanetworkopen.2026.9347

Data
Additional Information: NCT04551872
Data available: No
